# Selective Electrochemical Conversion of Glycerol to Glycolic Acid and Lactic Acid on a Mixed Carbon-Black Activated Carbon Electrode in a Single Compartment Electrochemical Cell

**DOI:** 10.3389/fchem.2019.00110

**Published:** 2019-03-13

**Authors:** Ching Shya Lee, Mohamed Kheireddine Aroua, Wan Ashri Wan Daud, Patrick Cognet, Yolande Pérès, Mohammed A. Ajeel

**Affiliations:** ^1^Department of Chemical Engineering, Faculty of Engineering, University of Malaya, Kuala Lumpur, Malaysia; ^2^Laboratoire de Génie Chimique, Université de Toulouse, CNRS, INP, UPS, Toulouse, France; ^3^Centre for Carbon Dioxide Capture and Utilization (CCDCU), School of Science and Technology, Sunway University, Bandar Sunway, Malaysia; ^4^Department of Engineering, Lancaster University, Lancaster, United Kingdom; ^5^Department of Chemistry, Al-Karkh University of Science, Baghdad, Iraq

**Keywords:** glycerol, electro-oxidation, electro-reduction, lactic acid, glycolic acid

## Abstract

In recent years, the rapid swift increase in world biodiesel production has caused an oversupply of its by-product, glycerol. Therefore, extensive research is done worldwide to convert glycerol into numerous high added-value chemicals i.e., glyceric acid, 1,2-propanediol, acrolein, glycerol carbonate, dihydroxyacetone, etc. Hydroxyl acids, glycolic acid and lactic acid, which comprise of carboxyl and alcohol functional groups, are the focus of this study. They are chemicals that are commonly found in the cosmetic industry as an antioxidant or exfoliator and a chemical source of emulsifier in the food industry, respectively. The aim of this study is to selectively convert glycerol into these acids in a single compartment electrochemical cell. For the first time, electrochemical conversion was performed on the mixed carbon-black activated carbon composite (CBAC) with Amberlyst-15 as acid catalyst. To the best of our knowledge, conversion of glycerol to glycolic and lactic acids via electrochemical studies using this electrode has not been reported yet. Two operating parameters i.e., catalyst dosage (6.4–12.8% w/v) and reaction temperature [room temperature (300 K) to 353 K] were tested. At 353 K, the selectivity of glycolic acid can reach up to 72% (with a yield of 66%), using 9.6% w/v catalyst. Under the same temperature, lactic acid achieved its highest selectivity (20.7%) and yield (18.6%) at low catalyst dosage, 6.4% w/v.

## Introduction

Glycolic and lactic acids are hydroxyl acids consisting of carboxyl and alcohol groups. Glycolic acid is extensively used as a chemical exfoliator or antioxidant in the cosmetic industry. Similarly, lactic acid also shows broad applications in cosmetic and pharmaceutical industries. Considering the increasing demand of both acids in the cosmetic industry, the international market of lactic and glycolic acids has grown rapidly and is expected to reach USD $382 million (Research and Markets, 2015) and USD $2.78 billion (Grand_View_Research, 2016) by 2020, respectively.

Previous studies demonstrated that glycolic and lactic acids can be synthesized from a glycerol oxidation process (Kumar et al., 2008; Zhou et al., 2008; Lakshmanan et al., 2013; Purushothaman et al., 2014). The product variation is extremely reliant on the catalyst structure, especially the porosity of the catalyst support, and the type of metal catalyst and its particle size. Additionally, reaction conditions such as reaction temperature, the acidity or basicity of the medium and mole ratio of metal to substrate are also key factors that could influence the product selectivity (Katryniok et al., 2011; Bagheri et al., 2015; Wang et al., 2015). Table 1 shows the product selectivity and yield of glycolic and lactic acids which were obtained through the catalytic reaction from glycerol. From the study carried out by Lux et al., lactic acid was produced through a hybrid process of combining the electrochemical and catalytic process. In this approach, glycerol was initially converted to dihydroxyacetone and glyceraldehyde via electrochemical oxidation and then be catalytically converted to lactic acid. While the product selectivity of lactic acid is high, this process required complicated reaction set-up (Lux et al., 2010). In view of the work carried out by Lux et al., a single compartment electrochemical process is presented in this study in order to convert lactic acid from glycerol in a single step reaction.

**Table 1:** The product yield and selectivity of glycolic and lactic acids; and glycerol conversion attained from the previous catalytic approaches.

**Type of catalyst**	**Conditions**	**Glycerol conversion (%)**	**Lactic acid**		**Glycolic acid**		**References**
			**Y (%)**	**S (%)**	**Y (%)**	**S (%)**	
Au/C (1% CB)	T: 333 K P O_2_: 1 Mpa	100			–	40	Demirel-Gülen et al., 2005
Au/C (5% CB)	Time: 3 h NaOH medium	100			–	36	
Au/C	T: 323 K P O_2_: 0.3 Mpa	90			–	17.0	Dimitratos et al., 2006
Pd/C	NaOH medium	90			–	5.6	
Pt	T: 333 K	40			–	22	Rodrigues et al., 2013
Pt/C	P O_2_: 0.3 Mpa	81			–	19	
Au/C	Time: 3 h	61			–	18	
Pt/C-Au/C	NaOH medium	98			–	20	
Ptt/S-CNFs	T: 333 K P O_2_: 0.4 MPa Time: 6 h	89.9			–	8.9	Zhang et al., 2015
Au/PUF (295 nm)	T: 333 K	30			–	76	Gil et al., 2014
Au/PUF (236 nm)	P O_2_: 0.5 MPa	30			–	74	
Au/PUF (111 nm)	Time: 1 h	30			–	50	
Au/PUF (138 nm)	NaOH medium	30			–	55	
Pt/MCNN amount: 3.3 g	T: 333 K P O_2_: 0.3 MPa	88.5			–	12.1	Wang et al., 2015
N amount: 2.7 g	Time: 4 h	63.1			–	6.3	
AuPd/C + Mg(OH)_2_	Au: Pd = 1: 1.85 Catalyst = 1% wt T: 60°C Time: 4 h P O_2_: 3 bar	40			–	10	Fu et al., 2018
AuPd/C + NaHCO_3_	T: 60°C	30			–	10	
Bi-AuPt/Ac	O_2_ flow: 15 ml/min	31.5				14.3	Motta et al., 2018
Pt/C (NaOH)	T: 473 K P H_2_: 4 MPa Time: 5 h	20 92 (5 h)	–	0.62 0.48			Maris and Davis, 2007
Ru/C (NaOH)		20 100 (5 h)	–	0.47 0.34			
Pt/C (CaO)		30 100 (5 h)	–	0.58 0.58			
Ru/C (CaO)		20 85 (5 h)	–	0.54 0.48			
Alkaline metal-hydroxide KOH NaOH LiOH	T: 573 K Time: 1.5 h	>90 >90 >90	90.0 87.1 81.2	– – –			Shen et al., 2009
Au-Pt/TiO_2_	T: 363 K P: 0.1 MPa NaOH medium	30	–	85.6			Shen et al., 2010
Ir-based catalyst	T: 433 K P N_2_: 0.1 MPa Time: 15 h KOH medium	34.8	–	>95			Sharninghausen et al., 2014
Au-Pt/nCeO_2_	T: 373 K P O_2_: 0.5 MPa Time: 30 min NaOH medium	99	–	80			Purushothaman et al., 2014
Au/CeO_2_	T: 363 K P O_2_: 0.1 MPa NaOH medium	98	–	83			Lakshmanan et al., 2013
Ru/La_2_O_3_	T: 453 K P H_2_: 5 MPa Time: 10 h	31.5	–	8.5			Feng et al., 2014
Ru/MgO		60.4	–	21.3			
Ru/CeO_2_		85.2	–	3.1			
Pt/C	T: 363 K P O_2_: 0.1 MPa Time: 6 h LiOH medium	100	–	69.3			Zhang et al., 2016
Pd/C	T: 503 K P O_2_: 0.1 MPa Time: 3 h NaOH medium	99	–	68			Arcanjo et al., 2016
Pt/C		99	–	74			
Ir(NHC-PhSO_3_)(CO)_2_	T: 115°C Time: 3 h (microwave)		–	91			Finn et al., 2018
	Time: 24 h (conventional)		–	8			
Pt/ZnO	T: 260°C P: 46 atm Time: 30 h		60	60			Bruno et al., 2018

*T, Temperature; P, Pressure; atm, atmospheric; Y, Yield; S, Selectivity*.

Furthermore, as compared to the catalytic process that is usually conducted under high temperature and high pressure conditions, this study has focused on the electrochemical approach, which is performed over a new electrode: the mixed carbon-black activated carbon electrode (CBAC). The electrochemical process is a simple and robust process which can operate under low reaction temperature and ambient pressure. In agreement to the study by Zhou et al. (2018), electrochemical valorization of glycerol offers an absolutely green route to produce high added-value compounds. They studied a series of electrocatalyst from graphene nano-sheet supported Pt to oxidize glycerol to glycolic acid. A maximum selectivity of 65.4 % glycolic acid was obtained at applied potential 0.2 V (Zhou et al., 2018). Dai et al. (2017) studied the electrochemical conversion of glycerol to lactic acid on AuPt nanoparticle. At potential 0.45 V, the selectivity for lactic acid was up to 73%. In another study, Lam et al. (2017) produced lactic acid from glycerol over cobalt-based oxidative catalyst under galvanostatic mode with 43% of selectivity.

Although electrochemical conversion of glycerol to glycolic and lactic acids has been previously explored (Fashedemi et al., 2015; Hunsom and Saila, 2015; Saila and Hunsom, 2015; Dai et al., 2017; Lam et al., 2017; Zhou et al., 2018), all the studies deployed expensive materials for working electrodes, such as gold, platinum, palladium etc. In this study, the electrode material (activated carbon) consumed is greener and more cost effective compared to the noble metal electrode. The cost of the noble metal and activated carbon (per gram) are presented in Table 2 (Sigma_Aldrich^1^). The new carbon-based cathode electrodes (CBAC) were investigated by the author in an earlier study to produce 1,2-propanediol (1,2-PDO) from electro-reduction of glycerol. The selectivity of 1,2-PDO was reported as high as 86 % on CBAC electrode (Lee et al., 2017). Due to the high selectivity and reduction in material costs in the first attempt (Lee et al., 2017), this new electrode is proposed in the present study. The effect of catalyst dosage and reaction temperature will be explored and the reaction mechanism is proposed.

**Table 2:** The price of noble metal and activated carbon (adapted form Lee et al., 2017).

**Type of material**	**Price (USD $/g)**	**% Purity**	**CAS number**
Platinum^**^	2015.00	99	7440-06-4
Palladium^**^	1260.00	99	7440-05-3
Rhodium^**^	506.00	99	7440-16-6
Gold^**^	347.00	99	7440-57-5
Activated carbon^*^	0.11	99	7782-40-3

** *In the form of nanopowder*.

* *In the form of powder*.

## Methodology

### Electrode Preparation

In this work, similar carbon electrode was prepared as reported by Lee et al. (2017) in her first study on the electro-reduction of glycerol (Lee et al., 2017). This carbon electrode was used as the cathode electrode in this current effort. The CBAC electrode (with geometrical surface area of 3.5 cm^2^) was prepared by blending 20 wt.%. carbon black (99% purity, specific surface area of 550 m^2^/g and average particle size of 13 nm; Alfa-chemicals, Malaysia) and 80 wt.% activated carbon (99.5% purity, specific surface area of 950 m^2^/g and average particle size of 100 μm,; Sigma Aldrich^1^) to a total weight of 1.0 g.

Later, 80% v/v 1,3-propanediol and 20% v/v polytetrafluoroethylene were added into the pre-mixed carbon powder to form a liquid-to-powder proportion of 2:1. The slurry was hard-pressed carefully to a round shape mold and dried in the oven based on the subsequent heating program: 373 K (2 h), 453 K (1 h), 523 K (1 h), and lastly 623 K for 30 min to allow the powder to dry completely and improve the electrode rigidity (Ajeel et al., 2015). The appearance of the electrode was characterized by scanning electron microscopy (SEM) equipped with an energy-dispersive E-ray (EDX) analyse (Hitachi SU-8000, Japan). The active surface areas of the electrode was acquired from the Cottrell equation as follows,

(1) $$\begin{array}{l} I=\;\frac{nFAD^{1/2}C_{0}}{\pi^{1/2}t^{1/2}} \end{array}$$

where *I* is the current (A), *D* is the diffusion coefficient (6.20 × 10^−6^ cm^2^/s), *F* is the Faraday constant 96487 (C/mol), *A* is the active surface area of the electrode (cm^2^), *t* is the time (s), n is the number of electrons and *C*_0_ is the bulk concentration of K_4_Fe(CN)_6_ (mol/cm^3^).

### Electrochemical Conversion

**Figure 1:**
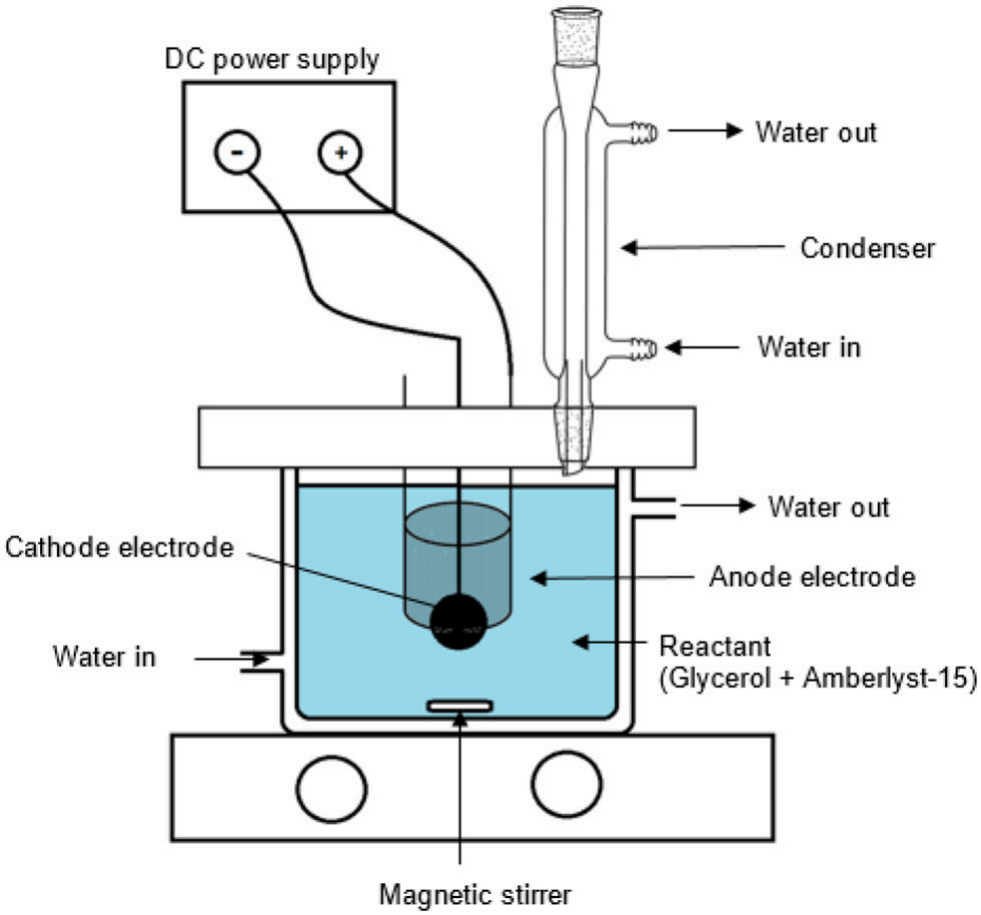
Electrochemical set-up (single compartment reactor).

Electrochemical experiment was carried out in a single compartment electrochemical cell as shown in Figure 1. The cell was filled with 0.1 L of 0.30 M of 99% purity glycerol solution and the reaction was performed over Pt anode electrode (geometrical surface area: 33 cm^2^) and CBAC cathode electrode (geometrical surface area: 3.5 cm^2^) for 8 h. A constant current at 2.0 A was supplied to the system by a DC power supply. In this study, Amberlyst-15 was used as an acid catalyst. The acid catalyst was investigated by the author in an earlier study. It showed that the strong sulfonic acid group in the Amberlyst-15 can enhance the glycerol conversion, product selectivity and yield (Lee et al., 2017). The effect of catalyst dosage (6.4, 9.6, and 12.8% w/v) and reaction temperature [room temperature (300 K), 323 and 353 K] were studied.

### Characterization and Quantification of the Electrochemical Conversion

The products obtained were characterized by gas chromatography-mass spectroscopy (GC-MS) (Agilent Model 7890, United States) and quantified by gas chromatography (Agilent Model 6890, United States) equipped with a flame ionization detector (FID). Compounds were separated by a ZB-Wax column (30 m × 0.25 mm × 0.25 mm) (Phenomenex, United States). The obtained chromatograms were compared with the MS library and chemical standards. Glycerol, glycolic acid and lactic acid were analyzed using the following procedure: the front inlet temperature was controlled at 240°C. Initially, the oven temperature was fixed at 45°C and maintained for 5 min. Later, it was ramped at 10°C/min to reach 240°C at the final temperature and maintained for another 5 min. The sample injection was 1 μL. Glycerol conversion, product selectivity as well as yield were calculated based on the Equations (2–4), respectively.

(2) $$\begin{array}{l} Glycerol\;conversion\;\left(\%\right)=\;\frac{Gycerol\;converted\;\left(C\;mole\right)}{Total\;glycerol\;in\;reactant\;\left(C\;mole\right)}\;\times \;100\;\% \end{array}$$

(3) $$\begin{array}{l} Product\;selectivity\;\left(\%\right)=\;\frac{Product\;\left(C\;mole\right)}{Total\;of\;all\;products\;in\;liquid\;phase\;\left(C\;mole\right)}\;\times \;100\;\% \end{array}$$

(4) $$\begin{array}{l} Product\;yield\;\left(\%\right)=\;\frac{Product\;\left(C\;mole\right)}{Total\;glycerol\;in\;reactant\;\left(C\;mole\right)}\;\times \;100\;\% \end{array}$$

## Results and Discussion

### Electrochemical Conversion

**Figure 2:**
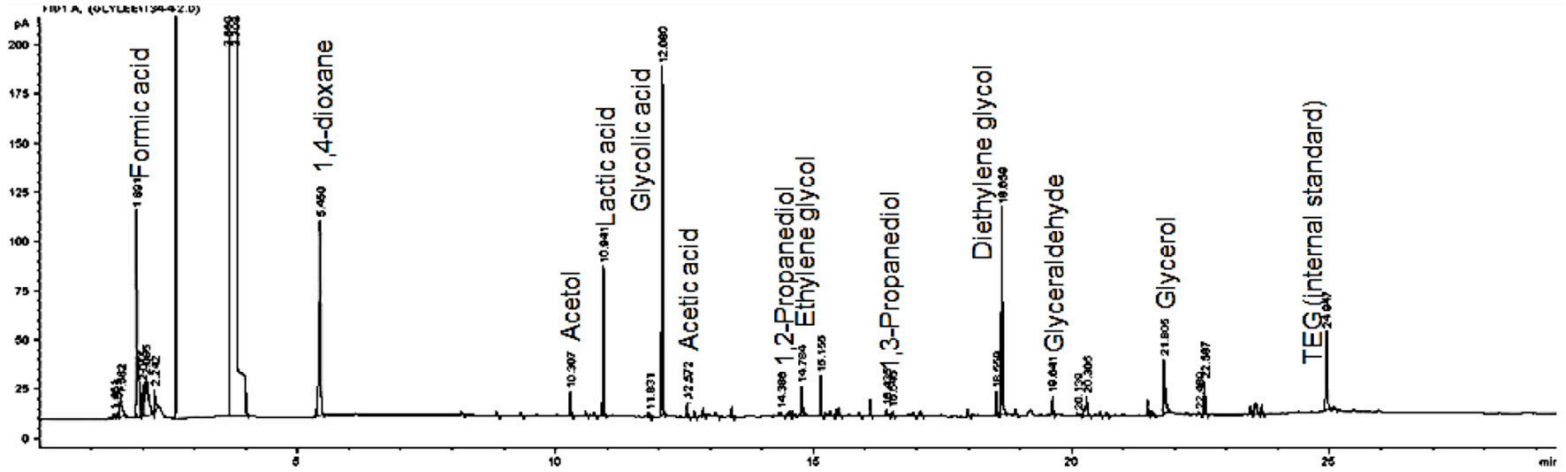
GC chromatogram of the products obtained from the electrochemical conversion of glycerol.

Based on the GC chromatogram as shown in Figure 2, the main compounds obtained from this study are glycolic acid and lactic acid. Other compounds i.e., ethylene glycol, acetic acid, formic acid, acetaldehyde, 1,3-propanediol (1,3-PDO), glyceraldehyde, acetol and 1,2-propanediol (1,2-PDO) were produced in small amount.

### Reaction Mechanism

In the electrochemical study, glycerol can possibly be oxidized via one of two pathways namely glyceraldehyde pathway or dihydroxyacetone (DHA) pathway. Based on a review published in 2012, the pathway's option can be determined by a few parameters, i.e., electrode material, applied potential, and pH of the reaction medium. In this case study, DHA pathway was involved (Simões et al., 2012). However, DHA was undetectable in the GC analysis. Whereby, it might have gone through the enolization process in the acidic condition (Van De Vyver et al., 2015), and further oxidized into pyruvic acid (PA).

**Figure 3:**
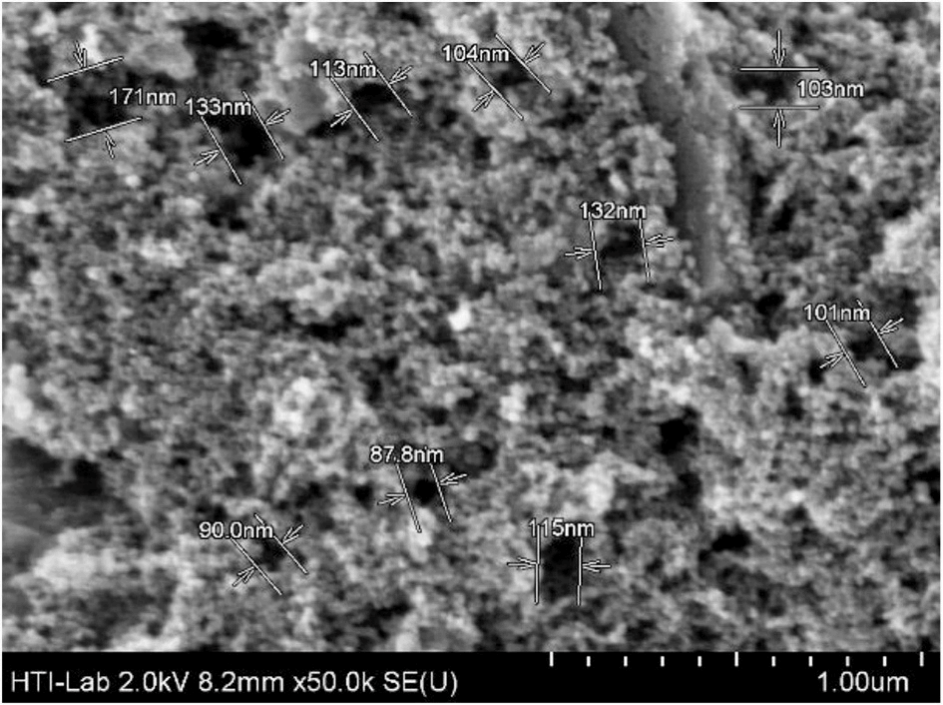
SEM image of CBAC electrode (pore sizes: 90−170 nm).

**Scheme 1:**
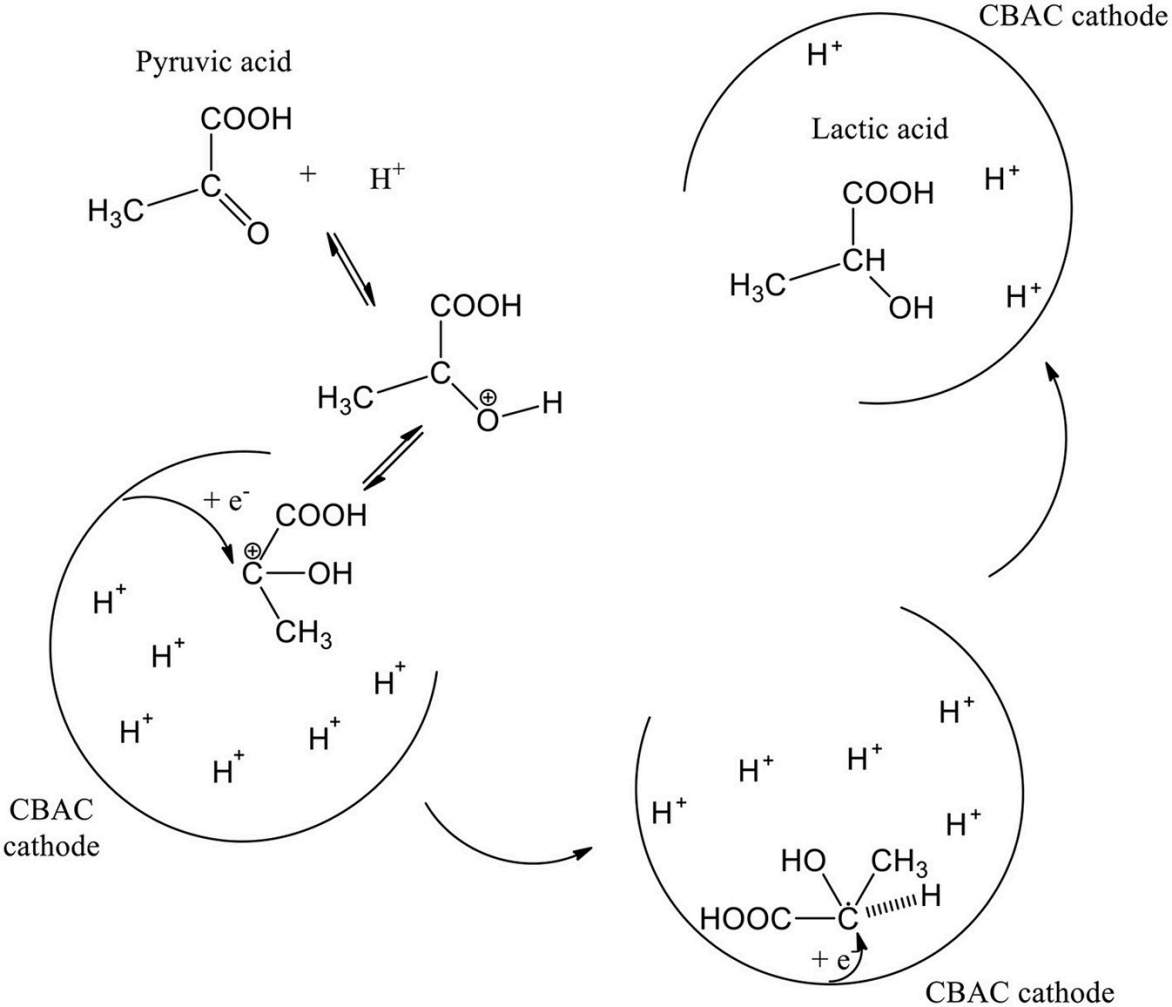
Formation of lactic acid on the porous surface of CBAC cathode electrode.

**Scheme 2:**
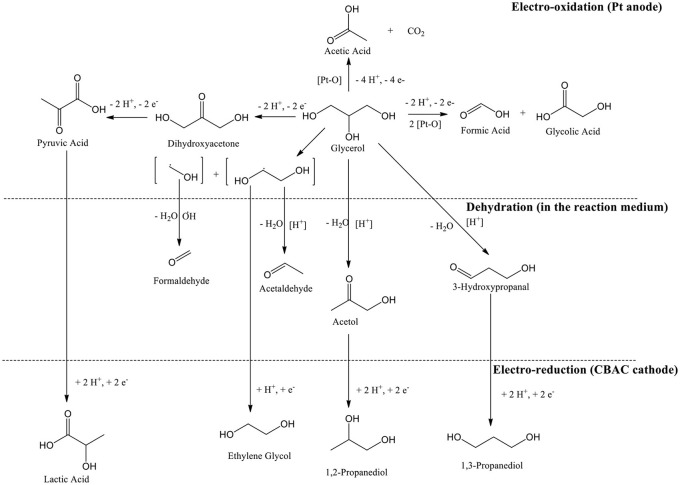
Proposed reaction mechanism of the overall electrochemical conversion for glycerol.

Since the electrochemical process was studied in a single compartment; lactic acid can be formed straightway via electroreduction of PA (Martin et al., 2005, 2006), and so PA was not detected in the analysis too. In addition, the activated carbon-based cathode electrode that was specially prepared for this study is highly porous (SEM image in Figure 3), Intermediate compounds that were produced from oxidation and dehydration processes (e.g., hydroxypropanal, pyruvic acid, and acetol) could be trapped or held in the porous surface thus enhancing the electrochemical reduction process (Qi et al., 2014) (Scheme 1). 1,3-PDO and 1,2-PDO were most likely formed from the electroreduction of hydroxylpropanal and acetol, respectively (Hunsom and Saila, 2015). At the anodic region, glycolic acid was likely produced via oxidation of glycerol through C-C bond cleavage. When glycolic acid continued to oxidize, acetic acid could be formed (Gomes et al., 2013). The proposed mechanism is shown in Scheme 2.

### Effect of Catalyst Dosage

**Figure 4:**
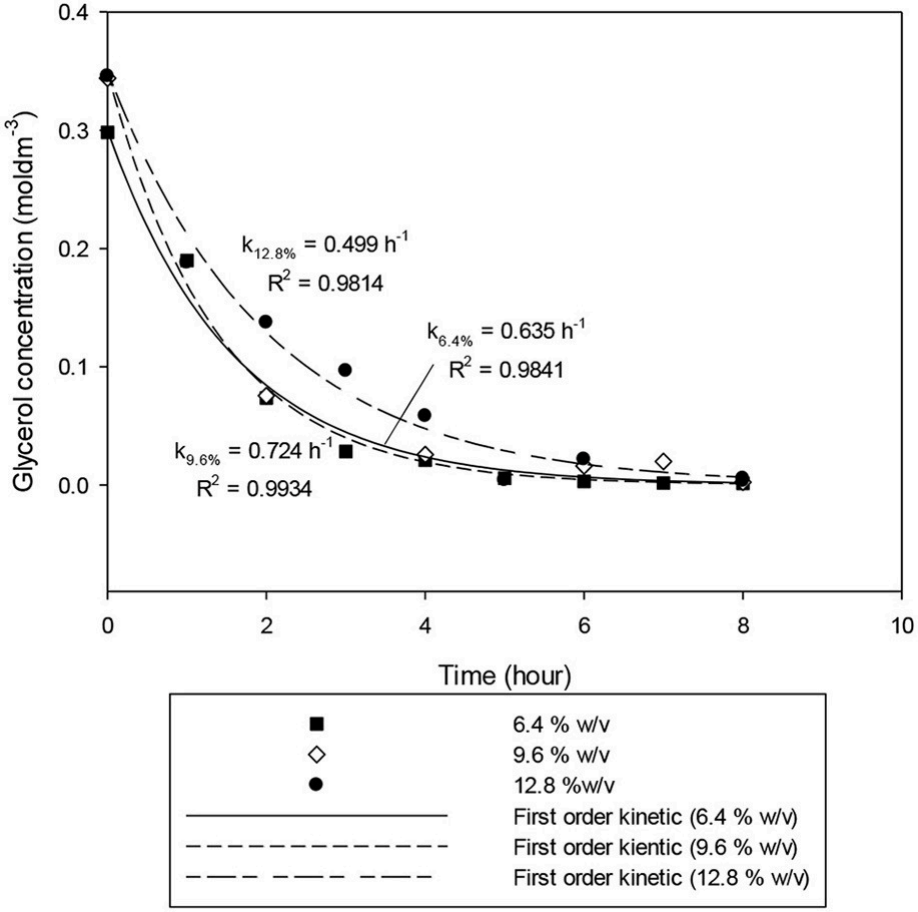
First-order kinetic model of the electrochemical conversion of glycerol at catalyst dosage ranging from 6.4 to 12.8% w/v. Other parameters such as temperature (353 k), and applied electric current (2.0 A) was maintained constant for 8 h.

**Figure 5:**
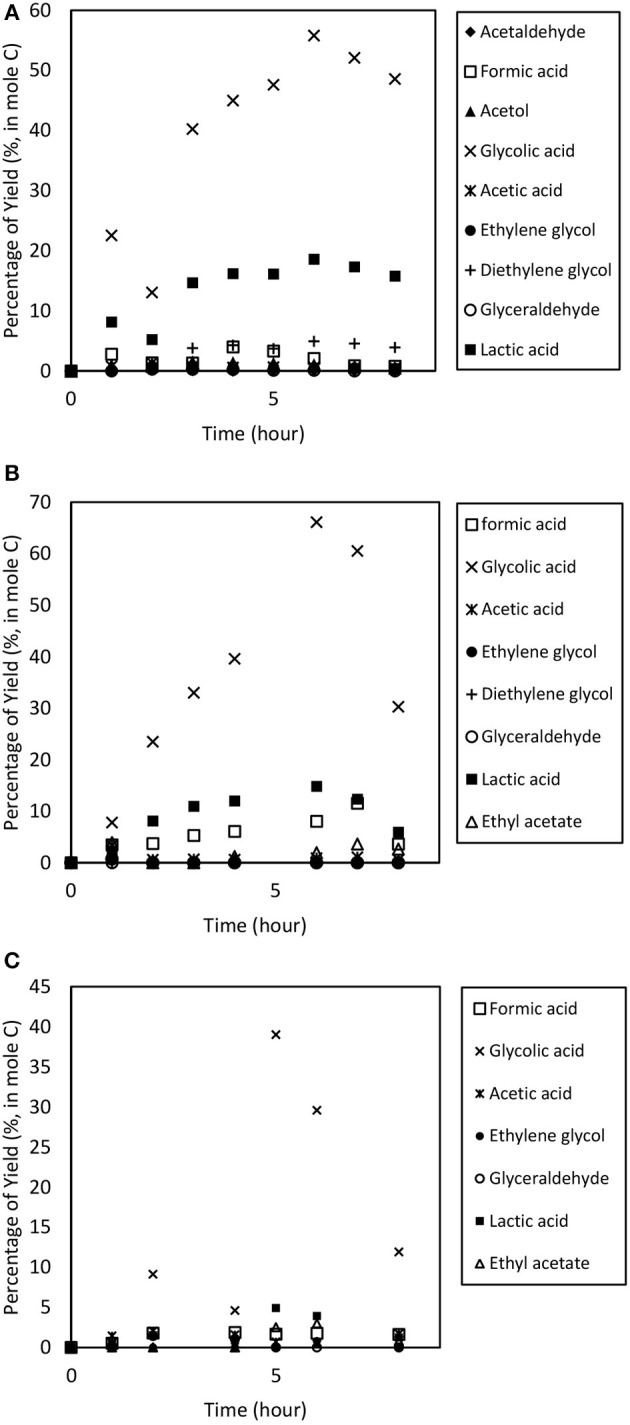
Product distribution of the electrochemical conversion of glycerol at catalyst dosage ranging from **(A)** 6.4, **(B)** 9.6 to **(C)** 12.8% w/v. Other parameters such as temperature (353 k), and applied electric current (2.0 A) was maintained constant for 8 h.

**Figure 6:**
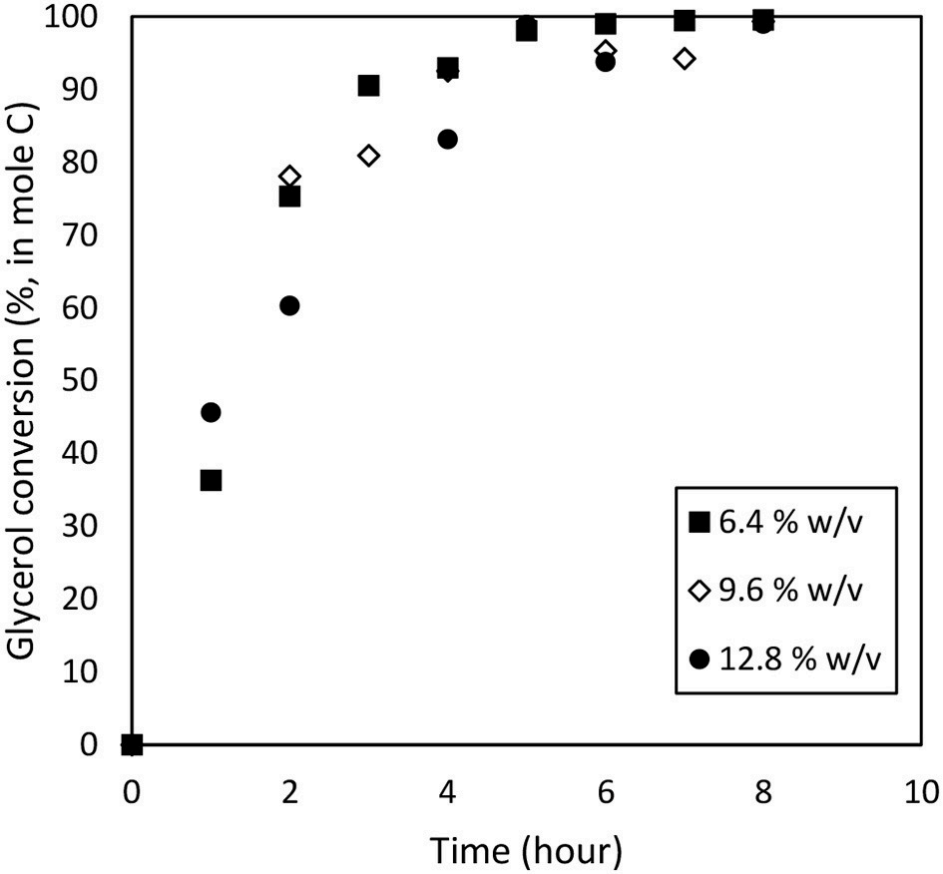
Glycerol conversion from the electrochemical conversion of glycerol at catalyst dosage ranging from 6.4 to 12.8% w/v. Other parameters such as temperature (353 k), and applied electric current (2.0 A) was maintained constant for 8 h.

To study the catalyst dosage for Amberlyst-15, the catalyst dosage was varied ranging from 6.4 to 12.8% w/v. Other parameters such as temperature (353 k), and applied electric current (2.0 A) were maintained constant for 8 h. The glycerol conversion rate is described in Figure 4. When the catalyst dosage increases, the conversion rate increased from 0.635 to 0.724 h^−1^. As seen in Figure 5, the product distributions varied when the catalyst dosage increased. The highest glycolic acid yield was achieved after 6 h with selectivity of 72.0%, using 9.6% w/v of catalyst. However, lactic acid preferred at low catalyst dosage (6.4% w/v), 18.6% of yield was obtained after 6 h of reaction. After a critical dosage of catalyst, the conversion slightly reduced. It can be attributed to the poisoning effect of catalyst. The products may compete with the reactant for the active sites thus causing a self-inhibitive effect, decreasing the conversion rate and yields (Bühler et al., 2002; Farma et al., 2013). In this case study, glycolic acid competed with glycerol and further oxidized into other compounds, for example CO_2_. Figure 6 illustrates the glycerol conversion. In all cases, overall the conversions were above 99%.

### Effect of Temperature

**Figure 7:**
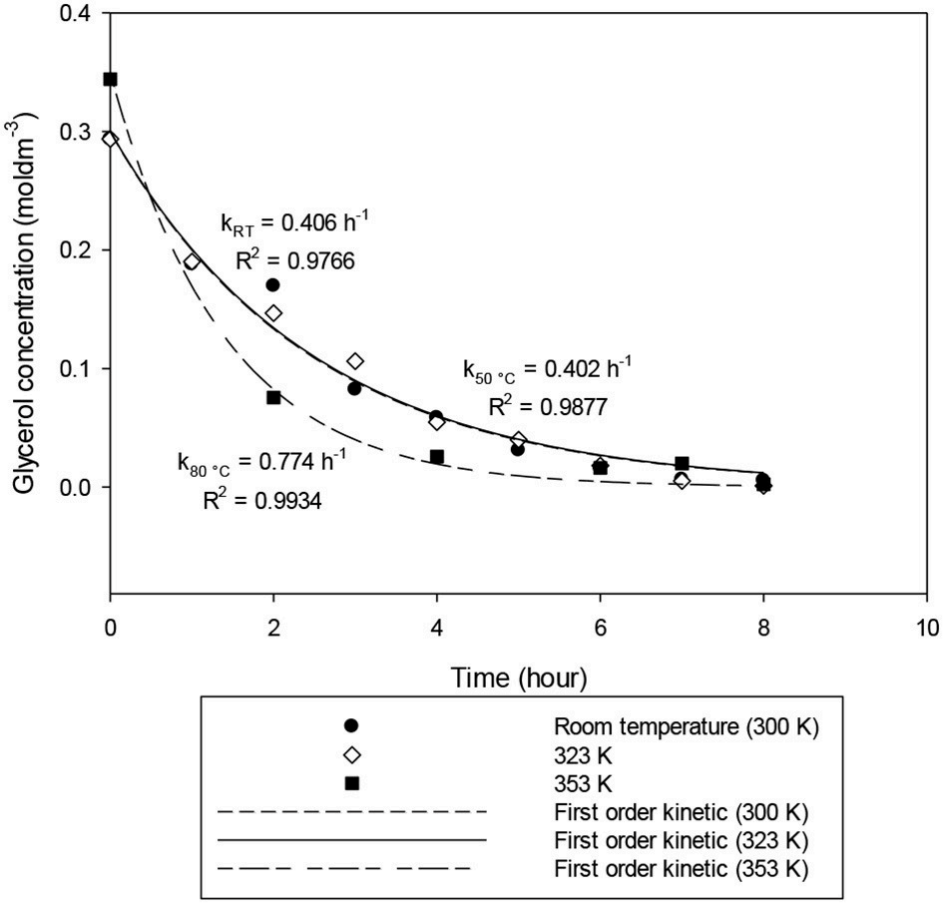
First-order kinetic model of the electrochemical conversion of glycerol at operating temperature ranging from room temperature (300 K) to 353 K. Other parameters such as applied electric current (2.0 A) and catalyst dosage (9.6% w/v) were maintained for 8 h.

To study the optimal temperature for electrochemical conversion, the temperature was varied ranging from room temperature (300 K) to 353 K, while keeping other variables constant at 2.0 A and 9.6% w/v of catalyst. Results depicted in Figure 7 show that the glycerol conversion rates increased with an increase in temperature. At 300 K and 323 K, the conversion rates were around 0.400 h^−1^. It increased to 0.724 h^−1^, when the temperature reached 353 K.

**Figure 8:**
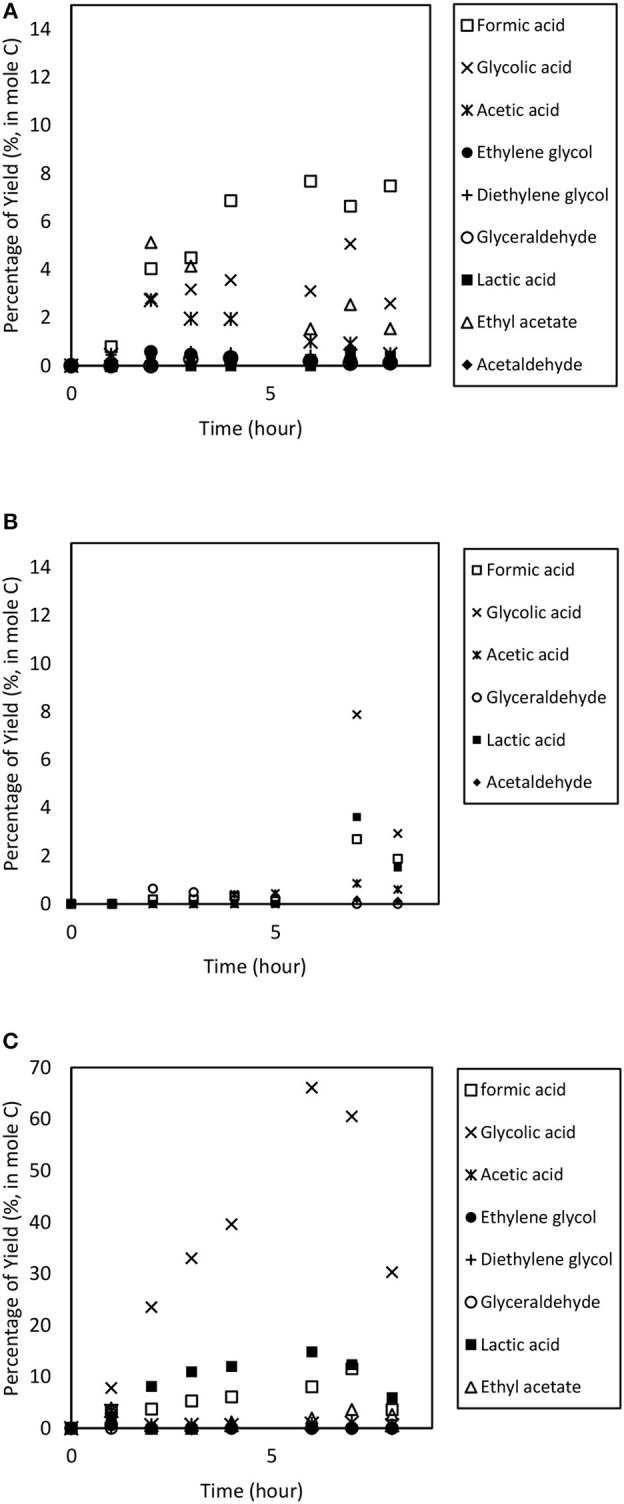
Product distribution from the electrochemical conversion of glycerol at operating temperature ranging from **(A)** room temperature (300 K); **(B)** 323 K to **(C)** 353 K. Other parameters such as applied electric current (2.0 A) and catalyst dosage (9.6% w/v) were maintained for 8 h.

**Figure 9:**
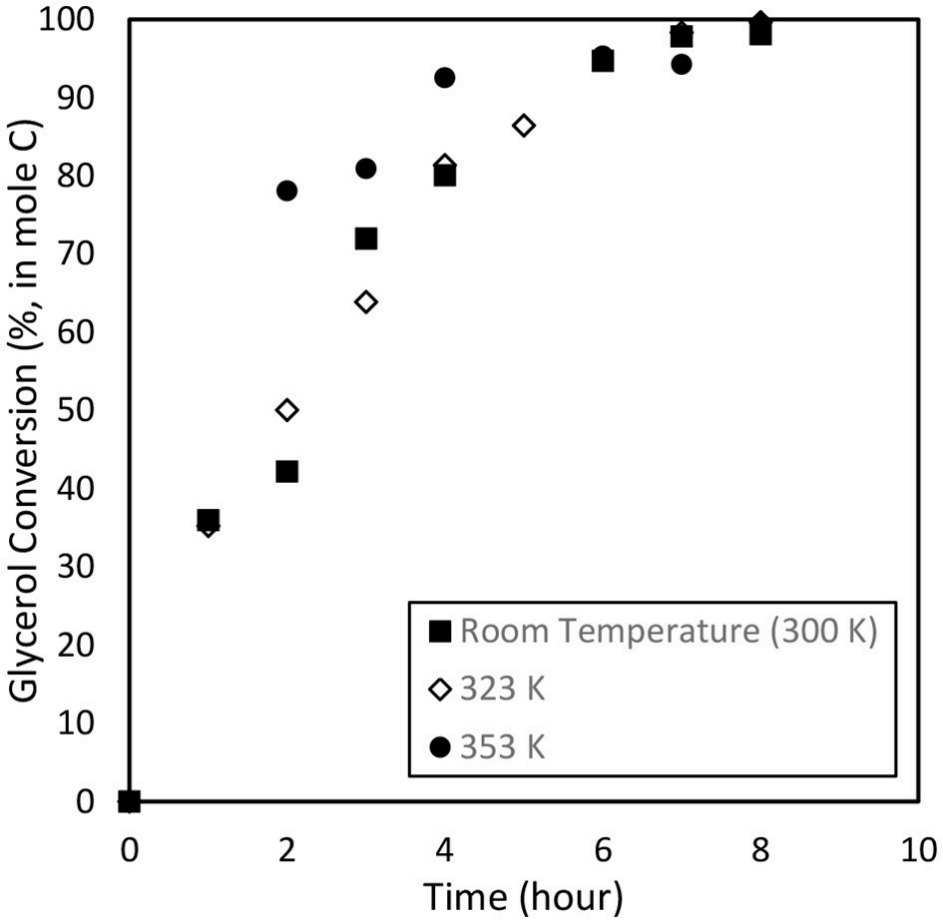
Glycerol conversion from the electrochemical study at operating temperature ranging from room temperature (300 K) to 353 K. Other parameters such as applied electric current (2.0 A) and catalyst dosage (9.6% w/v) were maintained for 8 h.

During the electrochemical process, a temperature increase accelerates the breakage of C-C bond, thus converting the glycerol into glycolic acid. Highest glycolic acid yield was achieved at 66.1% with selectivity of 72.0% after 6 h of reaction. In addition, an increase in temperature could also enhance OH adsorption on the Pt anode electrode thus reducing the barrier for O-H and C-H dissociations, and subsequently improving the oxidation performance (Beden et al., 1987; Yang et al., 2012; Zhang et al., 2012). Higher temperatures yielded higher production of lactic acid yield (14.8%). This could be due to the thickness of the diffusion layer which is effectively reduced (Gupta et al., 1984), thus improving the diffusion rate of intermediate compounds such as pyruvic acid to the CBAC cathode electrode which accelerates the formation of lactic acid. Figure 8 illustrates the products distribution for the three trials. Acetic acid and formic acid were found in all trials. Other compounds e.g., acetaldehyde, ethylene glycol, ethyl acetate and diethylene glycol were observed inconsistently. Overall, 90% glycerol conversion was achieved at the three temperatures, ranging from 300 to 353 K. The results are displayed in Figure 9.

### Energy Consumption

Energy consumptions in electrochemical conversion of glycerol were examined depending on the operating parameters such as catalyst dosage and temperature. The values were calculated using Equation (5):

(5) $$\begin{array}{l} E_{Gly\;Conv.}\;=\;\frac{iU\Delta t}{\left(C_{o}\;-\;C_{t}\right)V} \end{array}$$

Where, *E*_*Gly Conv*__._ is energy consumption in glycerol conversion (kWh/kg), i is current (A), *U* is voltage (V), Δ*t* is time (h), *C*_0_is initial glycerol concentration (g/L), *C*_*t*_ is final glycerol concentration (g/L), and *V* is volume (L).

The energy consumption values for glycerol conversion after 8 h of reaction at 2.0 A are tabulated in Table 3. Both parameters show similar energy consumed, in the range of 9–12 kWh/kg, due to the conversion of all trials are above 90%.

**Table 3:** Energy consumption in electrochemical conversion of glycerol depending on operating parameters.

**Operating parameters**	**Reaction conditions**	**Energy consumption of glycerol conversion (kWh/kg)**
Catalyst Dosage	Glycerol Concentration: 0.3 M Volume: 0.1 L Current: 2.0 A Catalyst: 6.4–12.8 % w/v Potential: 15.4–20.4 V	9.0–12.8
Reaction temperature	Glycerol concentration: 0.3 M Volume: 0.1 L Current: 2.0 A Catalyst: 300–353 K Potential: 15.4–16.8 V	9.0–12.7

### Research Outlook

The proposed electrochemical method resulted in a comparable or higher selectivity of glycolic acid with that previously reported in the studies tabulated in Table 1, which is about 72.0%. Nevertheless, the method proposed in this work is simpler, requiring at lower temperature and ambient pressure, which save energy and cost. The catalyst used can accelerate the reaction by enhancing the electron transfer between the electrolyte and electrode (Francke and Little, 2014), thus avoiding over-oxidation to other inauspicious by-products, i.e., acetic acid.

Based on the experimental results, lactic acid's yield and selectivity are lower as compared to the past published works (Table 1). Although the results are unpromising, the newly prepared in-house carbon-based electrode (CBAC electrode) appeared to be more cost-effective than the metal-based catalyst used in the reported chemical conversion studies (Arcanjo et al., 2016; Zhang et al., 2016). In accordance with (Qi et al., 2014) and Zhang et al. (2014), pore sizes is the key factor to stimulate the product selectivity, by controlling the activated carbon ratio in the upcoming trials the lactic acid selectivity could be boosted (Qi et al., 2014; Zhang et al., 2014).

Nevertheless, the main challenge for this work lies on separation and purification studies. This is always an important step in downstream operation to recover those valuable compounds produced from the reaction. The traditional separation methods include solvent extraction, crystallization, ion exchange, precipitation and acidification as well as adsorption. Nowadays, these methods become less popular because they hardly meet the modern green chemistry requirement (Anastas and Breen, 1997). Membrane technologies have attracted significant interests in recent years. Nano-filtration, electro-deionization, and electro-dialysis are the common separation methods that have been widely studied (Huang et al., 2007; González et al., 2008; Boontawan et al., 2011). Electro-dialysis which consists of a cation-selective membrane, an anion-selective membrane in a two-compartment cell is suggested for future product purification as it has been extensively reported in the previous literatures for recovery of pyruvate (Zeli and Vasić-Rački, 2005), glycine (Elisseeva et al., 2002), formic acid (Luo et al., 2002), lactate (Boniardi et al., 1996; Danner et al., 2000; Madzingaidzo et al., 2002; Hábová et al., 2004), and propionate (Fidaleo and Moresi, 2006).

## Conclusions

In this study, the single compartment electrochemical conversion for glycerol was examined. Glycerol was successfully converted to glycolic acid and lactic acid on the Pt anode electrode and the new activated carbon-based cathode electrode: CBAC electrode. Based on the optimization study, the experimental conditions favorable to glycolic acid production were a 353 K temperature with 9.6% w/v Amberlyst-15, leading to the highest yield of 66.1% and selectivity of 72.0%. Lactic acid was preferably generated at 353 K with the presence of 6.4% w/v Amberlyst-15. In this conditions, the highest yield obtained was 18.6% with selectivity of 20.7%. The highest glycerol conversions achieved were around 99%. These findings successfully provide a new route to convert glycerol to lactic acid via one step electrochemical process.

## Data Availability

All datasets generated for this study are included in the manuscript and/or the supplementary files.

## Author Contributions

All authors listed have made a substantial, direct and intellectual contribution to the work, and approved it for publication.

### Conflict of Interest Statement

The authors declare that the research was conducted in the absence of any commercial or financial relationships that could be construed as a potential conflict of interest.

## Footnotes

^1^Available online at: http://www.sigmaaldrich.com/ (Accessed July 1, 2016).
